# Factors associated with delayed initiation of breastfeeding in health facilities: secondary analysis of Bangladesh demographic and health survey 2014

**DOI:** 10.1186/s13006-021-00360-w

**Published:** 2021-01-22

**Authors:** Shahreen Raihana, Ashraful Alam, Tanvir M. Huda, Michael J. Dibley

**Affiliations:** 1 Sydney School of Public Health, Faculty of Medicine and Health, The University of Sydney, Sydney, New South Wales Australia; 2 Maternal and Child Health Division, International Centre for Diarrhoeal Disease Research, Bangladesh (icddr,b), Dhaka, Bangladesh

**Keywords:** Breastfeeding, Initiation, Bangladesh demographic and health survey 2014, Caesarean, Health facilities

## Abstract

**Background:**

Irrespective of the place and mode of delivery, ‘delayed’ initiation of breastfeeding beyond the first hour of birth can negatively influence maternal and newborn health outcomes. In Bangladesh, 49% of newborns initiate breastfeeding after the first hour. The rate is higher among deliveries at a health facility (62%). This study investigates the maternal, health service, infant, and household characteristics associated with delayed initiation of breastfeeding among health facility deliveries in Bangladesh.

**Methods:**

We used data from the 2014 Bangladesh Demographic and Health Survey. We included 1277 last-born children born at a health facility in the 2 years preceding the survey. ‘Delayed’ breastfeeding was defined using WHO recommendations as initiating after 1 h of birth. We performed univariate and multivariable logistic regression to determine factors associated with delayed initiation.

**Results:**

About three-fifth (*n* = 785, 62%) of the children born at a health facility delayed initiation of breastfeeding beyond 1 h. After adjusting for potential confounders, we found delayed initiation to be common among women, who delivered by caesarean section (adjusted Odds Ratio (aOR): 2.93; 95% CI 2.17, 3.98), and who were exposed to media less than once a week (aOR: 1.53; 95% CI 1.07, 2.19). Women with a higher body mass index had an increased likelihood of delaying initiation (aOR: 1.05; 95% CI 1.01, 1.11). Multiparous women were less likely to delay (aOR: 0.71; 95% CI 0.53, 0.96).

**Conclusions:**

Delayed initiation of breastfeeding following caesarean deliveries continues to be a challenge, but several other health facility and maternal factors also contributed to delayed initiation. Interventions to promote early breastfeeding should include strengthening the capacity of healthcare providers to encourage early initiation, especially for caesarean deliveries.

## Background

Global recommendations from WHO and UNICEF clearly state that all newborns are to be placed in ‘skin-to-skin contact’ with their mother immediately after birth to ‘support the initiation of breastfeeding within the first hour of birth [1, 2]. Early initiation of breastfeeding within the first hour of birth is a crucial step towards ensuring optimal breastfeeding practices sustained throughout infancy. It supports the bond between mother and child [3], increases chances of breastfeeding success, and lengthens the duration of breastfeeding [4]. Mothers who delay the initiation of breastfeeding beyond the first hour would often also tend to terminate breastfeeding sooner than recommended.

Recent systematic reviews [5, 6] and a global report by WHO and UNICEF [1, 7] suggest that delaying initiation of breastfeeding beyond the first hour of birth increases the risk of neonatal death by 33%. Studies in several low- and middle-income countries also report a 42–44% reduction in risk of death among newborns who initiated breastfeeding within the first hour of birth. Studies also suggest that timely initiation of breastfeeding enhances newborns immune system [8]. It is associated with reduced likelihood of mortality by protecting against infection [5, 9], sepsis and severe illnesses during early newborn [10], neonatal and infancy period.

The Baby Friendly Hospital Initiative and WHO [11] further emphasize that women would require assistance with positioning and attachment of the newborn to her breast. In circumstances when the mother has undergone a caesarean section, it is important to initiate breastfeeding as soon as the mother can respond [11, 12]. Mothers in the post-operative stage following a caesarean section at a health facility often require extra help [12] to position the newborn for its first suckle. Irrespective of the place of childbirth and mode of delivery, it is recommended to initiate breastfeeding before [1] the child has any routine procedures such as weighing, cord-cutting, bathing and wrapping.

Despite this evidence and guidelines emphasizing the importance of initiating breastfeeding early, the rate of early initiation of breastfeeding is still low in many developing countries. In Bangladesh, mothers exclusively breastfeed 55% of their infants under 6 months of age, a 13 percentage point increase over the past decade; and continue breastfeeding 87% of their infants until 2 years, a ten percentage point increase over the past decade. Only 51% of the newborns initiate breastfeeding within the first hour of birth [13, 14], which is a 27 percentage point increase from 2004 [15]. At the same time, institutional deliveries increased from 9% in 2004 to 39% in 2014. Yet, this increase has only translated into a modest decrease in the rate of delayed initiation of breastfeeding in Bangladesh [14, 15]. According to the WHO tool for assessing infant and young child feeding practices [11], the current prevalence of early initiation of breastfeeding in Bangladesh is far below 90%, the lower limit for “very good”. Nationally representative data of Bangladesh [14] also suggests that the rate of delayed initiation is higher among deliveries that take place at health facilities. In 2014, 62% of facility deliveries, and 41% of home deliveries, initiated breastfeeding after the first hour of birth [14], which appears contradictory to improved practices expected at health facilities.

Early initiation of breastfeeding, irrespective of the place and mode of delivery, is crucial in improving maternal and newborn health outcomes. However, there appears a gap in the practice to initiate breastfeeding within the first hour of birth, particularly among institutional deliveries in Bangladesh. This gap is an important area for research because we expect fewer women in these settings to experience delays as trained birth attendants should support them to initiate breastfeeding immediately after delivery. It is not clear which health service factors and maternal characteristics contribute to this consistent delay in breastfeeding initiation following delivery at a health facility. Such information can guide interventions to improve the maternal and newborn outcomes among institutional deliveries. The interventions required for home deliveries are likely to be very different for those occurring in facility deliveries. In this study, we investigate the factors associated with delayed initiation of breastfeeding among health facility deliveries in Bangladesh using data from the Demographic and Health Survey of Bangladesh, 2014.

## Methods

### Study settings

This study is a secondary analysis of data from the Bangladesh Demographic and Health Survey (DHS) 2014. This survey was conducted in 2013 and is a nationally representative survey that collected demographic and health-related data from 17,863 ever-married women aged 15–49 years from 17,300 households across Bangladesh. The DHS used a two-stage stratified sample of households from 600 enumeration areas (EA) (207 urban EA and 393 rural EA). The primary analysis performed using the DHS tools is reported in the Final Report of Bangladesh Demographic and Health Survey 2014 [14]. The DHS statistics manual [16] provides specific details of the sample design and data collection procedures. Data collection included administration of the *Household* and *Woman’s Questionnaire* to collect information on the respondent’s sociodemographic characteristics, birth history, and pregnancy and postnatal care. For this study, we restricted our sample size to women who delivered their last-born child born in the 2 years preceding the survey at a health facility.

### Definition of variables

#### Outcome variable

The outcome variable for this study is ‘delayed initiation of breastfeeding’ referring to any child for whom the mother, initiated breastfeeding after the first hour of birth. For this analysis, we coded delayed initiation of breastfeeding as a dichotomous variable of “within 1 hour” and “after 1 hour” for use in logistic regression models. We asked women who had a child born in the 2 years preceding the survey “How long after birth did you first put (child’s name) to the breast?” Women who responded ‘immediately’ or ‘within 0 or 1 h’ were coded as early initiators while all the rest we coded as delayed initiators. For this analysis to explore the determinants of delayed initiation, we have considered ‘delayed initiation’ as the outcome of interest. To examine the distribution of the outcome variable, we expanded the time to initiation of breastfeeding into four categories of ‘within 1 h’, ‘1-24 hours’, ‘24-48 hours’, and ‘≥ 48 hours’.

#### Exploratory variables

We selected the variables from potential confounders of early initiation of breastfeeding identified in prior studies from other countries. Also, variable selection depended on the availability of the relevant data in the Bangladesh DHS 2014 [14].

**Fig. 1:**
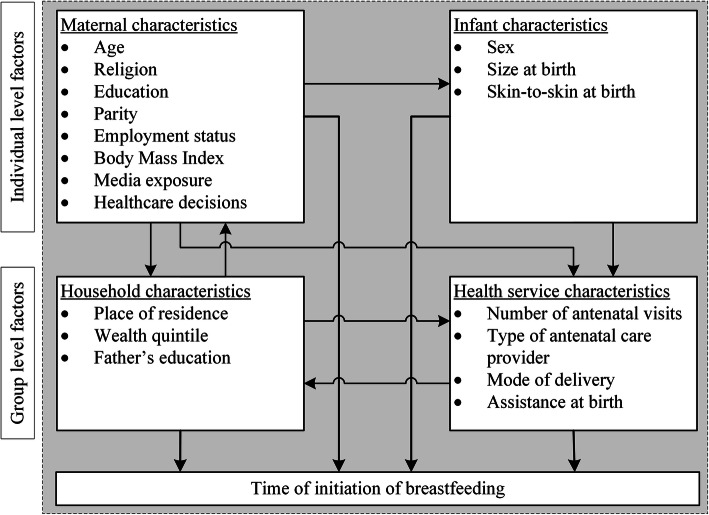
Conceptual framework of individual and group level factors associated with the time of initiation of breastfeeding among deliveries in a health facility. Adapted from frameworks presented in earlier studies [17–19] with some modification

The conceptual framework [17–19] (Fig. 1) summarises the selected potential individual and group level factors associated with the time of initiation of breastfeeding. We grouped these factors as i) maternal, ii) infant, iii) household, and iv) health service characteristics. We created categorical dummy variables by grouping continuous variables, using clinical and epidemiologic cut-offs. We treated the lowest group in each of the categories as the reference group to check the validity of the linear assumption. Group i) Maternal characteristics included mother’s age, religion, mother’s education, mother’s involvement in income generation activities, parity, body mass index, exposure to mass media, use of a mobile phone to access health services and responsible for healthcare decisions. Mother’s age was calculated by subtracting the child’s age from the mother’s age (self-reported) and recoded as < 20, 20–34 and 35–49 years. Islam is the most common religion in Bangladesh, and we recoded religion as ‘Islam’ and others. Mother’s education was categorized as higher, secondary, primary and no education with the higher education as the reference group. Involvement in income generation activities coded into a dichotomous variable as employed and not employed (self-reported). We coded parity as primiparous (first birth) and multiparous (more than one birth). We recoded the person responsible for making healthcare decisions for the mother as ‘woman herself’, ‘joint decision (including the woman)’, and ‘someone else (husband/family)’. We coded the extent of mother’s exposure to mass media as ‘at least once a week’ versus ‘less than once a week or not at all’. We used a dichotomous variable to record the mother’s use of the mobile phone to get health services or advice. We treated body mass index (BMI) of the women as a continuous variable to assist interpretation. Group ii) We included the following infant characteristics in the analysis: sex of the child, size of child at birth, skin-to-skin contact after birth. Data on the birthweight of the child is not available in Bangladesh DHSs. The alternative information collected in the survey is the mother’s perception of the size of the infant at birth which is a proxy for the objective measure of birth weight and often used in limited-resource settings [20]. For ease of interpretation, size of child at birth was coded as ‘average or larger’, ‘smaller than average’ and ‘very small’. Skin-to-skin contact is the act of putting a naked child on to the mother’s bare skin soon after birth. We created a dichotomous variable for whether the child had skin to skin contact immediately after birth. Group iii) Household characteristics included the father’s education, place of residence, and wealth index. We coded the father’s education, the same as the mother’s education. Place of residence indicated whether the mother lived in urban or rural parts of the survey area. Since the DHS does not collect information on household income and expenditure, household’ wealth index’ was used as a proxy indicator for socio-economic status. We calculated the wealth index score using the information on the household’s ownership of consumer goods household characteristics, source of drinking water, toilet facilities, and a few other factors related to the household’s socio-economic status. The asset index was then constructed using the principal component analysis (PCA) [21] and adjusted for the urban and rural populations [22]. The DHS, being a household survey, allowed limited scope of extracting variables from the supply end of the health service delivery system. Group iv) Some of the important health service variables from mother’s recall included in this analysis are the number of antenatal care (ANC) visits, type of ANC provider and type and assistance at delivery. We categorized the number of ANC visits as recommended by the WHO to facilitate interpretation of these practices. We coded number of ANC visits as no ANC visits, 1–3 visits, and ≥ 4 visits [23]. Type of antenatal care provider coded as medically trained and medically not-trained. We combined training status of care provider present during childbirth and the type of delivery into a single variable to indicate the service received by the women during delivery. We coded the combined variable as ‘normal delivery by trained attendant’, ‘normal delivery by untrained attendant’, ‘caesarean delivery (by a trained attendant)’.

### Statistical analysis

We examined delayed initiation of breastfeeding against a set of independent variables to explore the factors associated with delaying initiation beyond the first hour of birth. We used STATA version 15.0 (StataCorp, College Station, TX, USA) for all analyses. The analysis settings of the dataset were assigned using the command’ svyset’ to designate the sampling unit, survey weights and the cluster design of the survey. We calculated weighted frequencies for all potential explanatory variables and used descriptive statistics to explore the distribution of these factors, and used the binary outcome variable ‘delayed initiation’ for all logistic regression analysis. We applied univariate logistic regression to estimate the unadjusted association between the time of initiation of breastfeeding and potential explanatory factors. Then selected factors showing an independent association with *p* < 0.25 for the initial multivariable logistic regression model. We included several factors with *p* > 0.25 in univariate association in the initial model regardless of their level of significance as they were known to be associated with breastfeeding initiation. These factors included sex of the child, size of the child at birth, exposure to mass media, the person responsible for mother’s healthcare decision, assistance at birth, mother’s involvement in income generation activities. We constructed survey-weighted multiple logistic regression models to specify the association of all potential determinant variables as a function of a set of explanatory variables. To avoid non-convergence, we used the variance inflation factor to check for collinearity among variables included in the initial baseline model and further investigated for any strong associations among the variables by finding the correlation between continuous variables and by cross-tabulating categoric variables. Because of the moderate correlation (*r* = 0.6) between the mother’s and father’s education, we excluded the latter variable from the baseline model. We checked the continuous variable for body mass index for linearity assumptions. We removed non-significant variables that were not confounders or were not needed in the model using a backward elimination process, starting with the variable with the least significant association. The final multivariable model included some non-significant variables which were identified a priori to be important determinants of delayed initiation of breastfeeding. We report the adjusted OR with 95% CI for all variables and interpreted statistical significance as *p* < 0.05.

#### Ethical considerations

Data used in this study is publicly available and is de-identified for anonymity. We accessed the data through the DHS website (https://www.dhsprogram.com/data) after completion of the user’s agreement and approval for use. Procedures and questionnaires for standard DHS surveys are reviewed and approved by the Institutional Review Board (IRB) at ICF International. ICF IRB ensures that the survey complied with the U. S Department of Health and Human Services regulations for the protection of human subjects [14]. Informed consent was obtained from all participants before interviewing them for the survey.

## Results

**Fig. 2:**
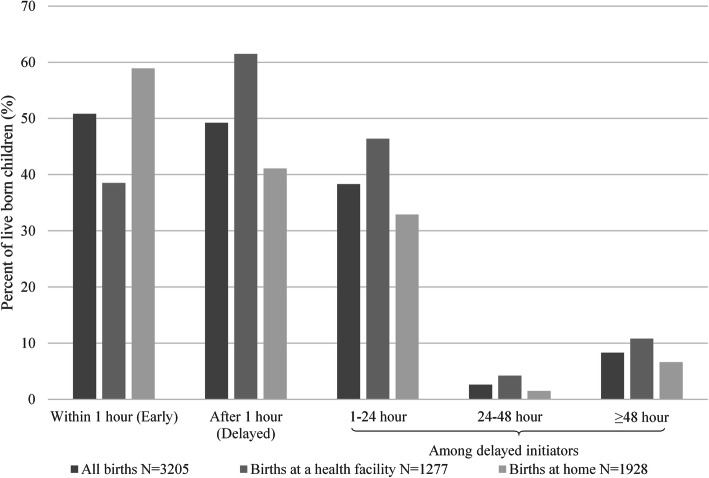
Initiation of breastfeeding among all births, facility births and home births

There were 3205 (weighted) last-born children (live births) born in the 2 years preceding the survey who had information on the time of initiation of breastfeeding, and 1277 were born in any health facility. For this analysis, we included all the 1277 children who were born to women aged 15–49 years at any health facility and had information on the time of breastfeeding initiation. Figure 2 presents the distribution of delayed initiation of breastfeeding among all births, births at a health facility or at home. A total of 492 (38.5%) children born at a health facility had their breastfeeding initiation delayed until after the first hour of birth.

The male to female ratio of the children born at a health facility was 55 to 45. The majority of the women were not employed (81%), were not placed on skin-to-skin contact with their newborn (71.4%) and gave birth through caesarean section (61.7%). The mean age of the mothers at the time of pregnancy was 24 ± 5 years, and 22% of the mothers were adolescents. As we analyzed women who delivered at a health facility, 94% of the mothers had been to at least one ANC visit, but only 48% of these women had been to all four recommended ANC visits. The mean body mass index of the women was 22.3 ± 4.1. Three in five women (64%) reported being able to take maternal healthcare decision either herself or jointly with her husband, and 11% were from the poorest wealth quintile.

Only 2.2% of the mothers never breastfed their child, and 92% of them were still breastfeeding at the time of the interview. The average time to initiate breastfeeding among all breastfed infants was 9.57 ± 2.7 h. However, the mean time to initiate breastfeeding among all infants delivered by a caesarean section was 13.12 ± 4.24 h, which is higher compared to children delivered through normal vaginal delivery assisted by a medically trained (3.99 ± 1.65 h) and medically untrained birth attendant (2.27 ± 3.58 h). The proportion of newborns with delayed initiation of breastfeeding was also higher for normal vaginal delivery by trained (46%) and untrained (33%) attendants in the health facilities compared to caesarean delivery (Table 1).

**Table 1:** Distribution of maternal, health service, infant and household characteristics among last-born children born the in the two years preceding the survey, 2014, *n* = 1277 (weighted), and among children with breastfeeding initiation delayed after birth by more than one hour, *n* = 785 (weighted)

Characteristics	Facility births ***n*** (weighted) = 1277, ***n (***%)	Breastfeeding initiation delayed, ≥ 1 h ***n*** (weighted) = 785, ***n*** (%)
**Maternal characteristics**		
Mother’s age		
15–19	284 (22.2)	187 (66.0)
20–29	786 (61.6)	463 (58.9)
30–39	194 (15.2)	125 (64.3)
40–49	12.9 (1.0)	9 (73.3)
Religion		
Islam	1173 (91.9)	720 (61.3)
Other	104 (8.1)	65 (62.8)
Mother’s education		
Higher	249 (19.5)	160 (63.9)
Secondary	699 (54.8)	439 (62.8)
Primary	251 (19.6)	142 (56.8)
No Education	78 (6.1)	44 (56.5)
Mother’s involvement in income-generating activity		
Employed	246 (19.3)	147 (59.7)
Not employed	1031 (80.7)	638 (61.9)
Parity		
Primiparous	636 (49.8)	408 (64.2)
Multiparous	641 (50.2)	377 (58.8)
The person who takes the mother’s healthcare-related decision		
Woman alone	128 (10.1)	71 (55.9)
Joint Decision	678 (53.6)	428 (63.2)
Someone else (husband/partner/family)	460 (36.3)	285 (60.5)
Body Mass Index	22.3 (±4.1)	22.6 (4.0)
Exposure to mass media		
At least once a week	872 (68.3)	531 (60.8)
Less than once a week or not at all	405 (31.7)	254 (62.8)
Use of mobile phone to get health service or advice		
Yes	553 (43.3)	375 (67.8)
No	724 (56.7)	409 (56.6)
**Health service characteristics**		
Number of ANC visits		
4+ ANC	611 (47.8)	396 (64.8)
1–3 ANC	593 (46.4)	345 (58.1
No ANC	73 (5.7)	44 (60.1)
ANC Provider		
Medically Trained	1075 (84.2)	657 (61.1)
Medically not-trained	202 (15.8)	128 (63.3)
Mode of delivery		
Normal	489 (38.3)	224 (45.9)
C-section	788 (61.7)	561 (71.2)
Assistance at birth		
Medically Trained	1254 (98.2)	777 (61.9)
Medically not-trained	23 (1.8)	8 (35.3)
Type and attendance at delivery		
NVD by a trained attendant	466 (36.5)	216 (46.4)
NVD by an untrained attendant	23 (1.8)	8 (33.3)
C-section	788 (61.7)	561 (71.2)
**Infant characteristics**		
Sex of child		
Male	696 (54.5)	422 (60.7)
Female	581 (45.5)	363 (62.4)
Size of the child at birth		
Average or Larger	1045 (81.8)	636 (60.8)
Smaller than average	145 (11.3)	97 (67.3)
Very small	87 (6.8)	52 (59.3)
The child put on skin-to-skin contact after delivery		
Yes	365 (28.6)	199 (54.4)
No	912 (71.4)	586 (64.3)
**Household characteristics**		
Paternal education		
Higher	323 (25.3)	200 (61.8)
Secondary	488 (38.2)	294 (60.2)
Primary	316 (24.7)	206 (65.4)
No Education	150 (11.8)	85 (56.8)
Place of residence		
Urban	502 (39.3)	316 (62.9)
Rural	775 (60.7)	469 (60.5)
Wealth quintile		
Lowest	139 (10.9)	86 (62.2)
Second	152 (11.9)	97 (63.9)
Third	216 (16.9)	123 (56.8)
Fourth	334 (26.1)	205 (61.4)
Highest	436 (34.2)	274 (62.7)

**Abbreviations:** *ANC* antenatal care, *NVD* normal vaginal delivery, *C-section* caesarean section

Table 2 shows the unadjusted and adjusted odds ratios of factors associated with delayed initiation of breastfeeding. Mothers who delivered through caesarean section had significantly higher unadjusted odds (OR = 2.87; 95% CI 2.10, 3.92) of delaying initiation compared to mothers who gave birth through normal vaginal delivery in the presence of a medically trained birth attendant. Once adjusted for the clustering effect of the survey design and the other potential confounders, the risk of delaying initiation remained significantly high and unchanged among the caesarean deliveries (aOR 2.93; 95% CI 2.17, 3.98). The likelihood of delayed initiation following caesarean delivery is higher than for women who had a normal vaginal delivery assisted by an untrained birth attendant. The results also suggest that women who were pregnant for the first time were more likely to delay initiation compared to those who had previously given birth (aOR 0.71; 95% CI 0.53, 0.96). Limiting exposure to any form of mass media to less than once a week or not at all is another likely risk factor (aOR 1.53; 95% CI 1.07, 2.19) of delaying initiation. Women with a higher body mass index also have higher odds of delaying breastfeeding initiation (aOR 1.05; 95% CI 1.01, 1.11). None of the infant and household characteristics was significantly associated with the time of breastfeeding initiation.

**Table 2:** Unadjusted and adjusted odds ratio of factors associated with breastfeeding initiation beyond the first hour of birth

Characteristics	Unadjusted OR	***P*** - value	aOR (95% CI)^**a**^	***P*** - value
**Maternal characteristics**				
Mother’s age				
15–19	1.00	0.34	–	
20–29	0.74 (0.53, 1.05)			
30–39	0.90 (0.55, 1.46)			
40–49	1.42 (0.30, 6.63)			
Religion			–	
Islam	1.00	0.88		
Other	1.03 (0.66, 1.62)			
Mother’s education			–	
Higher	1.00	0.60		
Secondary	0.98 (0.70, 1.36)			
Primary	0.75 (0.47, 1.22)			
No Education	0.76 (0.40, 1.42)			
Mother’s involvement in income-generating activity			–	
Employed	1.00	0.67		
Not employed	1.10 (0.70, 1.74)			
Parity				
Primiparous	1.00	0.06		
Multiparous	0.78 (0.61, 1.01)		0.71 (0.53, 0.96)	0.02
The person who takes the mother’s healthcare-related decisions			–	
Woman alone	1.00	0.40		
Joint Decision	1.33 (0.87, 2.03)			
Someone else (husband/partner/family)	1.21 (0.77, 1.91)			
Body Mass Index	1.05 (1.01, 1.08)	0.02	1.05 (1.01, 1.11)	0.02
Exposure to mass media				
At least once a week	1.00	0.50		
Less than once a week or not at all	1.11 (0.83, 1.48)		1.53 (1.07, 2.19)	0.02
Use of mobile phone to get health service or advice			–	
Yes	1.00	0.01		
No	0.62 (0.45, 0.86)			
**Health service characteristics**				
Number of ANC visits			–	
4+ ANC	1.00	0.26		
1–3 ANC	0.77 (0.55, 1.06)			
No ANC	0.82 (0.45, 1.51)			
ANC Provider			–	
Medically Trained	1.00	0.65		
Medically not-trained	1.11 (0.70, 1.77)			
Mode of delivery			–	
Normal	1.00	0.00		
C-section	2.94 (2.16, 3.99)			
Assistance at birth			–	
Medically Trained	1.00	0.01		
Medically not-trained	0.34 (0.15, 0.77)			
Type and attendance at delivery				
NVD by a trained attendant	1.00	0.00		
NVD by an untrained attendant	0.59 (0.25, 1.42)		0.50 (0.20, 1.28)	0.00
C-section	2.87 (2.10, 3.92)		2.93 (2.17, 3.98)	
**Infant characteristics**				
Sex of child			–	
Male	1.00	0.62		
Female	1.07 (0.82, 1.39)			
Size of the child at birth			–	
Average or Larger	1.00	0.35		
Smaller than average	1.38 (0.89, 2.13)			
Very small	0.94 (0.55, 1.60)			
The child put on skin-to-skin contact after delivery			–	
Yes	1.00	0.01		
No	1.52 (1.11, 2.12)			
**Household characteristics**				
Paternal education			–	
Higher	1.00	0.72		
Secondary	0.96 (0.71, 1.30)			
Primary	1.18 (0.78, 1.78)			
No Education	0.83 (0.44, 1.57)			
Place of residence			–	
Urban	1.00	0.54		
Rural	0.92 (0.69, 1.22)			
Wealth quintile			–	
Lowest	1.00	0.84		
Second	1.07 (0.58, 1.97)			
Third	0.79 (0.37, 1.67)			
Fourth	0.95 (0.57, 1.59)			
Highest	1.00 (0.61, 1.66)			

^a^Adjusted for religion, mother’s education, mother’s involvement in income-generating activities, number of antenatal visits, type of antenatal care provider, the person who takes the mother’s healthcare decision, sex of the child, size of child at birth, child put on skin-to-skin after delivery, place of residence, and wealth index

**Abbreviations:** *aOR* adjusted odds ratio, *OR* odds ratio, *ANC* antenatal care, *NVD* normal vagina delivery, *C-section* caesarean section

## Discussion

The prevalence of delayed initiation of breastfeeding beyond the first hour of birth was 61.5% among the newborns of the mothers who delivered at health facilities, which is high given that trained healthcare providers attended these births. The odds of delaying initiation of breastfeeding were significantly higher for children born by caesarean section, first-time mothers, women not exposed to any form of media, and women who had a higher overall body mass index. Findings in this study highlight the need for program managers to design interventions facilitating improved breastfeeding initiation practices among women who deliver at a health facility. Such targeted interventions would be particularly important for Bangladesh and other developing countries where institutional deliveries have increased, and majority of the institutional deliveries are through caesarean section. The findings showed that delayed initiation of breastfeeding beyond the first hour of birth was more likely with caesarean delivery. The 2014 Bangladesh Demographic and Health Survey reported that 37% of all deliveries occurred at a health facility. Of these deliveries 6 out of every 10 were via caesarean section. In the same year, 23% of all newborns were delivered by caesarean section, which was an increase from only 4% in 2004 [14]. Even though a third of all deliveries occurred at a facility, the majority of them resulted in a caesarean section. Thus, it is important to address the breastfeeding initiation practices in a post caesarean section setting. Three out of five children in this study population were born through caesarean section, of whom 71% delayed initiation beyond the first hour of birth. Delayed initiation was also higher for normal vaginal delivery assisted by trained and untrained providers at the health facilities. Although not a specific focus of this analysis, the proportion of delayed initiation among home deliveries (*n* = 1928), was similar when attended by either trained (44%) or untrained (41%) birth attendants.

A recent study [2] has established that caesarean delivery extends the time to initiation of breastfeeding because of several health facility-level factors. Women who deliver through caesarean section are also more likely to be unconscious after childbirth as a result of anaesthesia. Recommendations from World Health Organizations have suggested that in circumstances when the woman is unable to initiate breastfeeding as a result of medical procedures during childbirth, the newborn must be put to the breast as soon as the woman is conscious. If the woman received general anaesthesia during caesarean section, she could only initiate breastfeeding when she was awake and able to respond [24]. If the woman received regional anaesthesia during caesarean section, she could initiate breastfeeding even before the effects of the anaesthesia had worn off [25, 26]. This approach would ensure that the first feeding occurred within the recommended time when she was alert and not in severe pain.

Our study uses data from a large nationally representative cluster survey that used a standardized methodology for collecting the data. We explored the factors associated with delayed initiation of breastfeeding among hospital deliveries using a sub-sample of the surveyed population. There are few studies from Bangladesh (and South Asia) that examine the factors associated with the time of breastfeeding initiation. A study by Islam et al. [27] found a higher likelihood of delaying initiation of breastfeeding in deliveries at health facilities. Our study has extended these findings by focusing just on the group of women who deliver at a health facility to identify the factors associated with delayed initiation of breastfeeding in that setting. The global recommendations for institutional deliveries if followed, should lead to lower rates of delayed initiation, but this does not appear to be the case in Bangladesh and other countries in South Asia [1]. Our study helps understand why these institutions are still failing to comply with the global recommendations for early initiation of breastfeeding.

An important limitation of using data from a DHS survey is its retrospective nature. The data is from a recall period of up to 5 years. Or it is collected after the occurrence of the event of interest. For the breastfeeding initiation variable, there was a recall period of 2 years. It is likely the mothers of severely ill newborns, and the mothers of newborns who died after being born alive would probably remember the time to initiation more accurately than mothers whose children were well. Secondly, breastfeeding initiation time was defined using the mother’s report at the time of the survey. During a hospital delivery, especially in instances when the delivery is by caesarean section, the mother may not have been conscious immediately after childbirth to report the events around that time, including the time to initiation. Therefore, we have generated four broad breastfeeding initiation time categories: initiation within 1 h of birth, initiation from the first hour until the end of the first day after birth; initiation on the second day after birth; and initiation on or after the third day after birth. Thirdly, due to a lack of detailed health facility indicators, it was not possible to explore the effect of the knowledge and training status of the healthcare providers who assisted the women during childbirth. To account for this limitation, we have used some broad proxy indicators for whether or not the birth attendant was medically trained and linked this with the type of delivery. Additional modifiable factors like complications during pregnancy and delivery, duration of gestation, preterm delivery and newborn complications could not be included in this analysis as this information was not collected in the Bangladesh DHS. Fourthly, the body mass index used in this analysis was collected at the time of the survey and may not reflect the woman’s BMI pre-pregnancy or during pregnancy.

The results of our study confirm prior findings that birth by caesarean section is a major barrier to timely initiation of breastfeeding among deliveries at a health facility. Several studies have explored the consequences and impact of caesarean deliveries on breastfeeding initiation. For most women, post-surgical pain and discomfort would have a negative influence on the timely initiation of breastfeeding [28, 29]. Women who have had medically induced labour and emergency caesarean section often experience prolonged labour. The stress associated with the difficult labour due to anaesthetics and labour inducing hormones [30–32], leads to a delay in mother-infant interactions and in the time to initiate breastfeeding [33]. Moreover, women who planned to give birth by elective caesarean delivery may not intend to breastfeed or are not comfortable with initiating the natural process of breastfeeding [29]. Often, delayed initiation following caesarean delivery is also associated with reduced suckling ability, insufficient milk supply [31, 33] and physiologic effects leading to delayed onset of lactogenesis [34].

One study [31] further suggested that the delayed initiation encountered by women who deliver by caesarean section at a health facility is preventable. One of the aims of the global approach of the Baby Friendly Hospital Initiative has been to raise the rates of early initiation. Within this initiative participating hospitals encouraged women to initiate breastfeeding immediately following birth, educated their medical staff on the importance of early initiation and consequences of delayed initiation, and enacted the policy of rooming-in of the mother and child [35]. Modification in some health facility practices has the potential to create an enabling environment to ensure the early initiation of breastfeeding. Strong leadership, staff training, all-staff participation, repeat education of medical staff and developing a strategic approach could be some of the strategies adopted in a health facility to promote timely initiation of breastfeeding. The revised BFHI [36] guidance recommends that even if mothers are not able to initiate breastfeeding within the first hour after birth, they should still receive support for skin-to-skin contact and to breastfeeding as soon as they are able.

We further found maternal body mass index (BMI) to be significantly associated with delayed initiation of breastfeeding. The mean BMI of all women in this analysis was 22.3 (± 4.1). The analysis indicates that for every unit increase in BMI, the likelihood of delaying initiation increases six times. Our findings confirm the results of earlier studies [37, 38] that reported a delayed ‘onset of lactation’ among women with high BMI. One study [37] found that mothers with BMI > 27 kg/m^2^ were more than twice as likely to delay initiation than mothers with BMI < 27 kg/m^2^. Another study [38] reported that for every one-unit increase in prepregnant BMI, there was a delay in the onset of lactation by 0.5 h. A systematic review [39] looking at the relationship between maternal obesity and breastfeeding initiation presents the decrease in breastfeeding initiation rates among obese women, compared to women with normal weight and BMI. Most studies [40–42] attributed the possible association between high BMI and delayed breastfeeding initiation to anatomical or physiological, psychological and cultural factors. Obese and overweight women tend to have mechanical difficulties of attaching the newborn to their breasts [41] and are likely to have obstetric complications often leading to caesarean deliveries [42].

Exposure to mass media in the form of listening to the radio, watching television and reading newspaper is linked to early initiation of breastfeeding [43]. Findings in this study suggest that exposure to any form of mass media once a week or more would to reduce the odds delaying breastfeeding initiation. A report by the BBC Media Action [44] reports in 2015 that 85% of the adult population in Bangladesh had access to television, and 42% had access to a radio. Moreover, 97% of Bangladeshi adults had access to a mobile phone. Such high levels of media access indicate that transmission of pro-breastfeeding messages via radio, television or a mobile phone platform could impact on the breastfeeding initiation practice by women and healthcare providers at health facilities alike. Studies in Bangladesh [45] and Vietnam [46] have found that combining a mass media campaign using multiple media platforms and interpersonal counselling positively impacted optimal breastfeeding practices.

## Conclusions

Over the last decade many programs in developing countries, including Bangladesh, have promoted birthing at health facilities. Further, caesarean delivery at a health facility is a valuable tool to save the lives of women and their newborns in emergency obstetric situations [29, 47]. However, as we see from this analysis, the overuse of caesarean delivery in Bangladesh is associated with a higher likelihood of delaying breastfeeding initiation. Research also suggests that delayed initiation is likely to increase the risk of mortality and morbidity, especially in the newborn [5] and early newborn period [6, 10, 19]. These findings suggest that it is important for health facilities to establish guidelines on immediate newborn care practices to ensure early initiation following caesarean delivery. There is a need for national-level policies to provide a supportive environment at health facilities. These policies should include clinical guidelines and protocols for the successful initiation of breastfeeding following caesarean delivery. Interventions like the BFHI [48] have already demonstrated how training and knowledge transfer interventions support appropriate breastfeeding practices in hospitals in low- and middle-income countries. There will be a need for a consultative process to develop relevant guidelines and to test their effectiveness before upscaling to hospitals across Bangladesh. Such interventions would further have the potential to successfully institutionalize improved breastfeeding initiation practices following hospital deliveries in other South Asian countries. Future research needs to help develop and test clinical guidelines for ensuring early initiation of breastfeeding in health facilities, especially following caesarean delivery.

## Data Availability

The datasets analysed during the current study are publicly available through the Demographic and Health Survey website [https://www.dhsprogram.com/data].

## Abbreviations

ANC: Antenatal care
BFHI: Baby Friendly Hospital Initiative
BMI: Body Mass Index
CI: Confidence Interval
DHS: Demographic and Health Survey
EA: Enumeration areas
IRB: Institutional Review Board
OR: Odds Ratio
PCA: Principal Component Analysis
UNICEF: United Nations Children’s Fund
WHO: World Health Organization
